# Supplementary material for: Astaxanthin Inhibits Diabetes-Triggered Periodontal Destruction, Ameliorates Oxidative Complications in STZ-Injected Mice, and Recovers Nrf2-Dependent Antioxidant System
Source: Nutrients. 2021 Oct 12;13(10):3575. doi: 10.3390/nu13103575 (PMC8537008; doi:10.3390/nu13103575)
Supplement: Supplementary file 1 [file nutrients-13-03575-s001.zip › nutrients-1395995-supplementary.pdf]

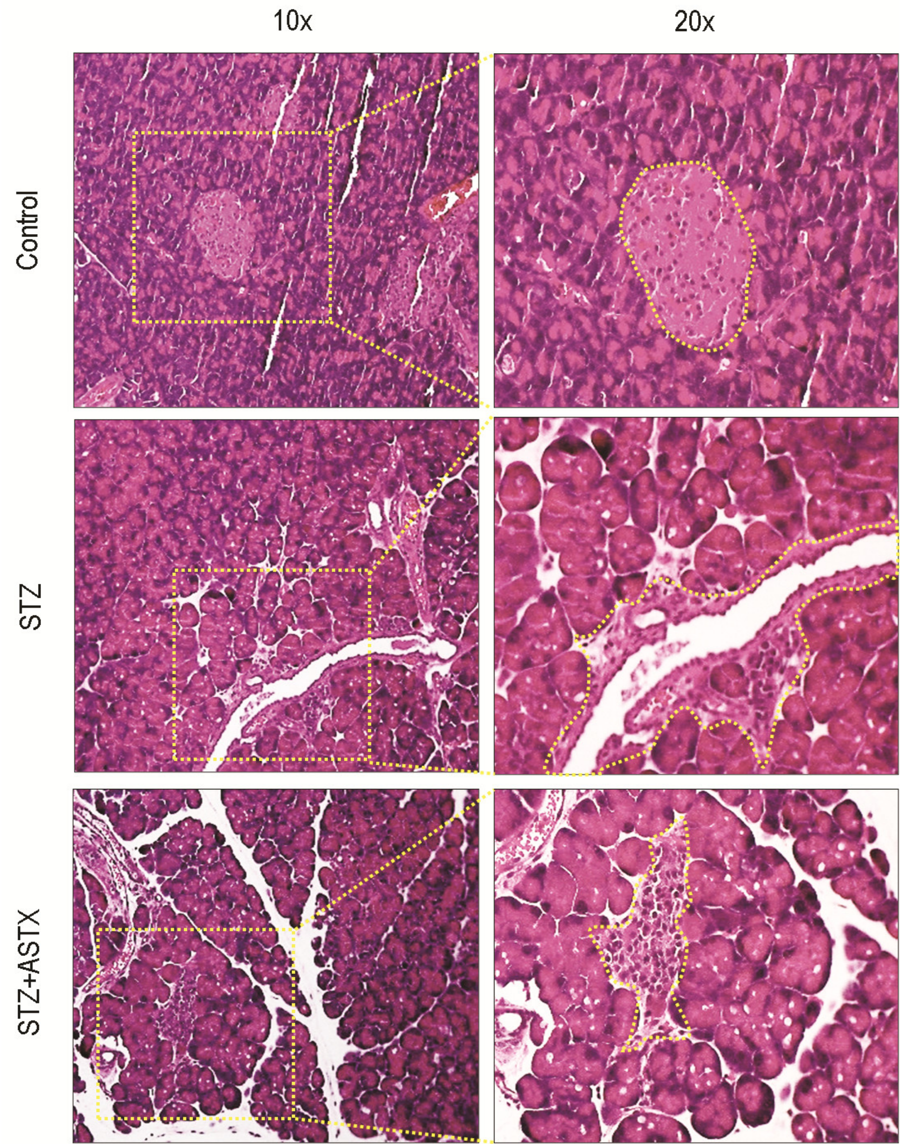

Figure S1. Oral supplementation with ASTX does not protect STZ-induced damage in pancreatic islets.

Pancreatic tissue sections were stained with H & E 60 days after the hyperglycemia induction. A representative result from five different samples is shown. The yellow dash lines indicate the pancreatic islets.

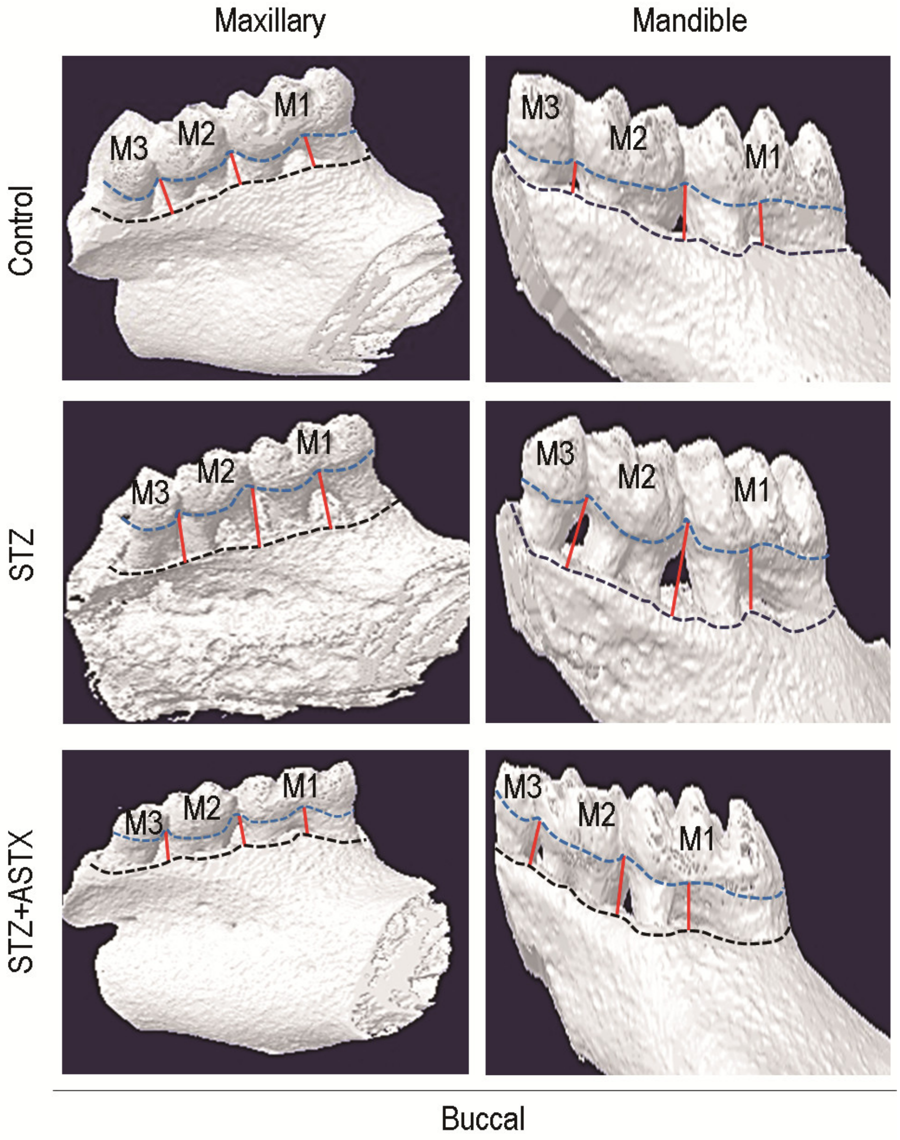

Figure S2. The 3D μCT images showing STZ-induced loss of alveolar bone at buccal region of maxillary and mandibular bones.

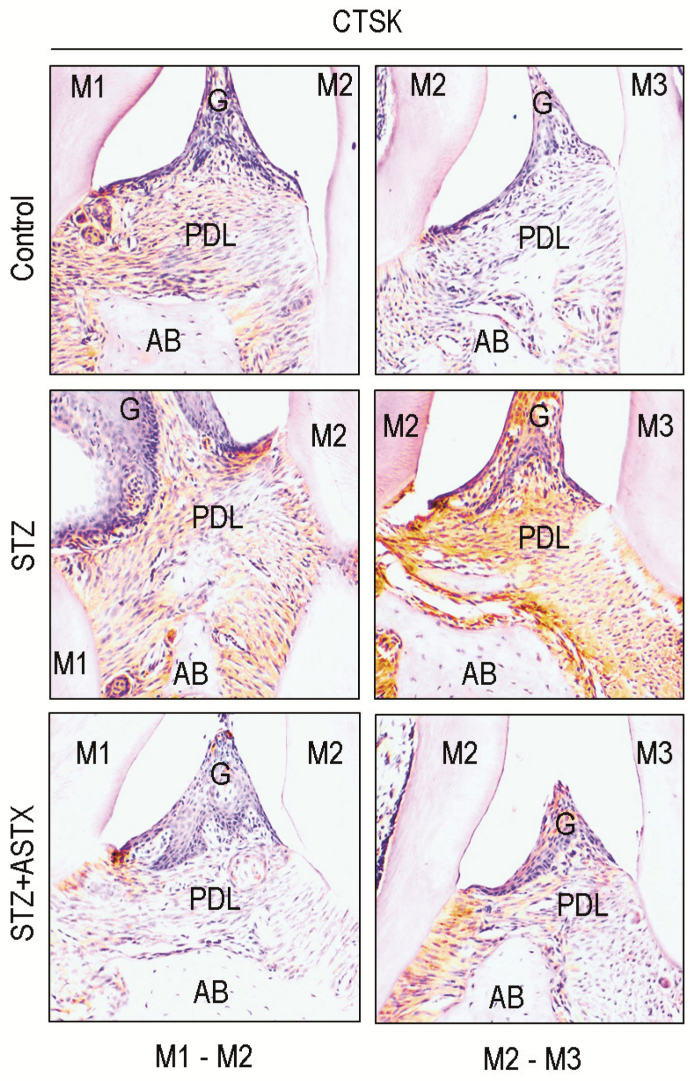

Figure S3. IHC staining showing the expression level of CTSK at the region of upper molars of mice groups 60 days after hyperglycemia induction (200× magnification).

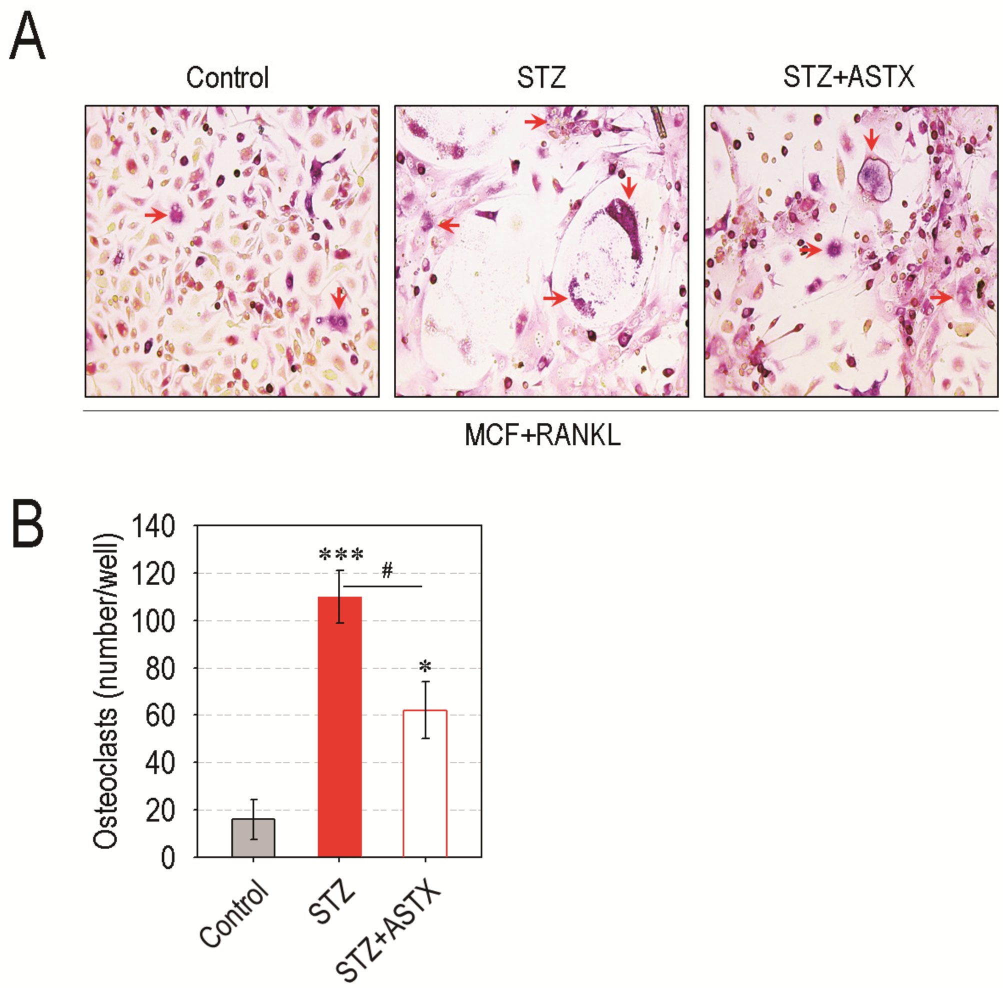

Figure S4. Bone marrow cells derived from STZ mice exhibit greater osteoclast forming activity than does the cells from control or STZ+ASTX mice.

The non-adherent bone marrow cells were isolated from mice groups 60 days post-hyperglycemia induction and incubated in the presence of M-CSF and RANKL for 7 days. (A) Photograph showing the TRAP-positive multinucleated cells and (B) the number of osteoclasts formed in the cultures. The red arrows indicate osteoclastic cells. $^{*}p < 0.05$ and $^{***}p < 0.001$ vs. control group; $^{\#}p < 0.05$ vs. STZ group.

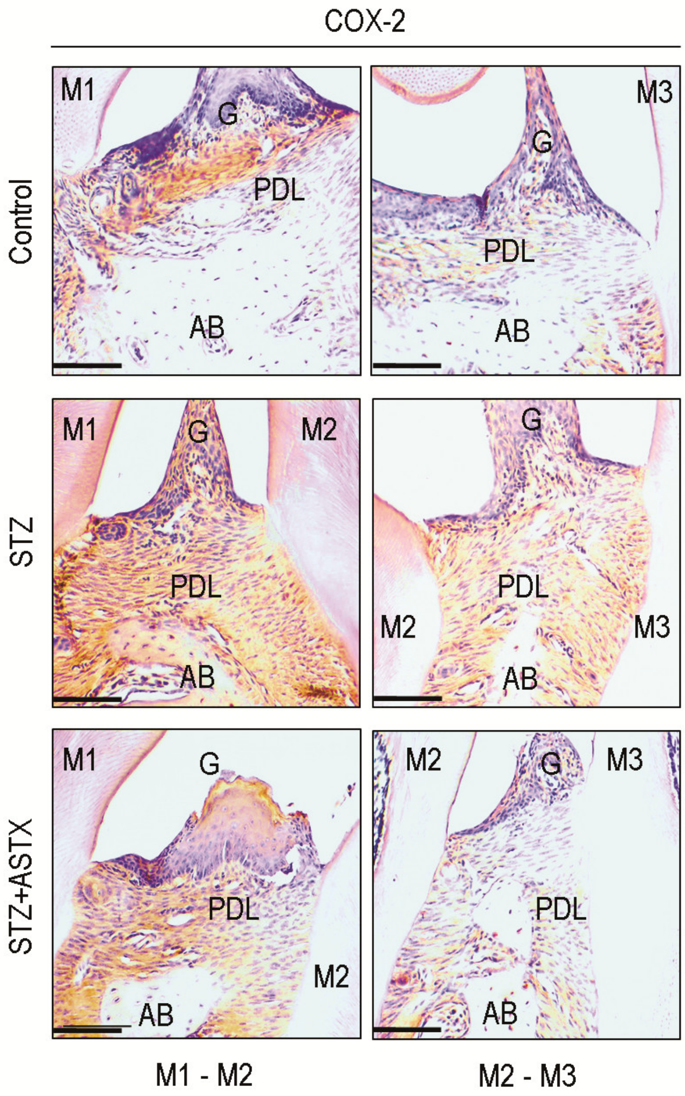

Figure S5. IHC staining showing level of COX-2 at the region of upper molars of mice groups 60 days after hyperglycemia induction (200× magnification/Bar = 100 μm).
